# A *Francisella tularensis* Schu S4 Purine Auxotroph Is Highly Attenuated in Mice but Offers Limited Protection against Homologous Intranasal Challenge

**DOI:** 10.1371/journal.pone.0002487

**Published:** 2008-06-25

**Authors:** Roger D. Pechous, Travis R. McCarthy, Nrusingh P. Mohapatra, Shilpa Soni, Renee M. Penoske, Nita H. Salzman, Dara W. Frank, John S. Gunn, Thomas C. Zahrt

**Affiliations:** 1 Department of Microbiology and Molecular Genetics and Center for Biopreparedness and Infectious Diseases, Medical College of Wisconsin, Milwaukee, Wisconsin, United States of America; 2 Department of Pediatrics, Medical College of Wisconsin, Milwaukee, Wisconsin, United States of America; 3 Center for Microbial Interface Biology, Department of Molecular Biology, Immunology and Medical Genetics, The Ohio State University, Columbus, Ohio, United States of America; Centre for DNA Fingerprinting and Diagnostics, India

## Abstract

**Background:**

*Francisella tularensis* is a Gram-negative coccobacillus that causes the febrile illness tularemia. Subspecies that are pathogenic for humans include those comprising the type A (subspecies *tularensis*) or type B (subspecies *holarctica*) biovars. An attenuated live vaccine strain (LVS) developed from a type B isolate has previously been used to vaccinate at-risk individuals, but offers limited protection against high dose (>1000 CFUs) challenge with type A strains delivered by the respiratory route. Due to differences between type A and type B *F. tularensis* strains at the genetic level, it has been speculated that utilization of an attenuated type A strain as a live vaccine might offer better protection against homologous respiratory challenge compared with LVS. Here, we report the construction and characterization of an unmarked Δ*purMCD* mutant in the highly virulent type A strain Schu S4.

**Methodology/Principal Findings:**

Growth of Schu S4 Δ*purMCD* was severely attenuated in primary human peripheral blood monocyte-derived macrophages and in the A549 human lung epithelial cell line. The Schu S4 Δ*purMCD* mutant was also highly attenuated in mice when delivered via either the intranasal or intradermal infection route. Mice vaccinated intranasally with Schu S4 Δ*purMCD* were well protected against high dose intradermal challenge with virulent type A or type B strains of *F. tularensis*. However, intranasal vaccination with Schu S4 Δ*purMCD* induced tissue damage in the lungs, and conferred only limited protection against high dose Schu S4 challenge delivered by the same route. The level of protection observed was similar to that conferred following vaccination with wild-type LVS or the analogous LVS Δ*purMCD* mutant.

**Conclusions/Significance:**

Collectively, these results argue that development of the next generation live attenuated vaccine for *Francisella* should be based on use of the less pathogenic type B biovar rather than the more reactogenic type A biovar.

## Introduction

*Francisella tularensis* is one of the most infectious bacterial species known, and is the etiological agent of the debilitating febrile illness tularemia. *F. tularensis* is comprised of four subspecies, of which subspecies *tularensis* (type A) and subspecies *holarctica* (type B) are the biovars most commonly associated with disease in humans. Type A strains cause the most severe disease manifestations, and are primarily found in North America. *F. tularensis* can be transmitted to humans from multiple reservoirs and via various infection routes [1]. Pneumonic tularemia resulting from the inhalation of aerosolized organisms is the most severe disease form, and has mortality rates approaching 30% to 60% when untreated [2], [3]. Due to its extreme virulence, ease of dissemination, low infectious dose, and previous weaponization, *F. tularensis* is considered a potential agent of bioterrorism. Currently, there is no licensed vaccine in the event of an intentional or accidental release of *F. tularensis*.

Attempts to develop a vaccine capable of inducing protective immunity against type A strains of *F. tularensis* have met with limited success. Early vaccine studies employed heat- or formalin- killed whole cell vaccines that offered limited efficacy with adverse side effects [4], [5]. A number of potential antigens have been identified and tested as components of subunit vaccines, but none aside from lipopolysaccharide have shown appreciable protective efficacy [6], [7]. A live vaccine strain (LVS) was developed in the former Soviet Union from multiple passages of the less pathogenic type B biovar, and remains the only available vaccine shown to offer significant protection against virulent *F. tularensis* strains. LVS, however, remains unlicensed in the United States as a vaccine for a number of reasons, and it remains fully virulent for many animals including mice. Early experiments involving human volunteers demonstrated that LVS given by scarification conferred protection against subsequent systemic type A challenge [3], [8]. LVS also protected against low-dose aerosol challenge (10–100 CFUs) with type A *F. tularensis*; however, it was unable to protect against a high dose aerosol challenge, the most likely dispersion route during a biological attack [2], [8]. Human studies involving respiratory vaccination with high doses of 10^6^–10^8^ LVS have demonstrated that this route of immunization enhances protective immunity against a large Schu S4 aerosol challenge (roughly 2500 CFUs) over that seen with LVS vaccination by scarification [9], [10]. Unfortunately, LVS was found to be more virulent to humans when given via aerosol, and in some cases the dose needed for protection resulted in tularemia, causing safety concerns for this delivery route [10]. Regardless, the ability of LVS to confer protection in humans demonstrates the potential for using live attenuated *Francisella* strains as vaccine candidates against tularemia.

Due to differences in genetic makeup and disease pathology between type A and type B *F. tularensis*, it has been predicted that respiratory vaccination with an attenuated type A strain of *F. tularensis* may offer the best protection against homologous aerogenic challenge [6], [7], [11], [12]. Until recently, construction of targeted mutations in this genetic background has been limited due to a lack of genetic tools and/or selectable markers. Recent advances have allowed a small number of targeted mutants to be generated and characterized in Schu S4, the prototypical type A *F. tularensis* strain [12]–[15]. These include a Schu S4 mutant deleted in the O-antigen locus *wbtDEF*. Although it is attenuated for growth in macrophages and in mice, this strain fails to protect mice following subcutaneous vaccination against moderate doses of wild-type SchuS4 delivered by the intraperitoneal route [14]. Similarly, deletion of either *iglC* or *FTT0918* attenuates growth of Schu S4 in macrophages and/or in mice [12]. However, intradermal vaccination with the Schu S4 Δ*iglC* mutant does not provide appreciable protective immunity, and the Schu S4 Δ*FTT0918* mutant retains a low level of virulence in mice following its administration by the intradermal route [12]. Finally, although a Schu S4 *dsbB* deletion mutant was found to be highly attenuated in mice following intranasal infection, intranasal vaccination with this strain failed to offer protection against challenge with even low doses (13 CFUs) of wild-type Schu S4 given by the same infection route [13]. Thus, none of the Schu S4 mutants generated to date have been found to confer protective immunity against high dose challenge with virulent type A strains of *F. tularensis*.

We recently reported that a marked deletion in an operon essential for purine biosynthesis (*purMCD*) in *F. tularensis* LVS resulted in a strain that was highly attenuated for virulence in murine macrophages and in mice when delivered by the intraperitoneal (i.p.) route of infection [16]. Furthermore, i.p. vaccination with this strain was capable of conferring protective immunity in mice following homologous challenge with wild-type LVS delivered by the same route [16]. In this study, an unmarked Δ*purMCD* mutant was generated in type A strain Schu S4 and its ability to confer protective immunity against wild-type *F. tularensis* was investigated. Growth of Schu S4 Δ*purMCD* was highly attenuated in primary human macrophages and in the human type II pneumocyte epithelial cell line A549. Schu S4 Δ*purMCD* was also highly attenuated in mice when delivered by the intranasal or intradermal routes of infection. However, intranasal vaccination of mice with the attenuated Schu S4 Δ*purMCD* mutant induced tissue damage in the lungs, and resulted in a protective immune response that was no better than that seen following vaccination with wild-type LVS or an analogous LVS Δ*purMCD* mutant. Given these results, we argue that the next generation *Francisella* vaccine should be based on the less pathogenic type B biovar.

## Results

### Construction and complementation of unmarked *purMCD* deletion mutants in *F. tularensis* LVS and Schu S4

The Δ*purMCD* mutation previously generated in LVS carried a kanamycin resistance marker for selection [16]. To alleviate concerns associated with the use of antibiotic resistance genes in type A *F. tularensis*, deletion of *purMCD* was performed in both LVS and Schu S4 without the incorporation of an antibiotic resistance marker. Potential unmarked *purMCD* mutants were screened for auxotrophy on Chamberlain's defined medium, and genetic deletions were confirmed by PCR and Southern blot hybridization (data not shown). There were no obvious growth defects of the mutant strains on complex media when compared to the wild-type parental strains (data not shown). Complementation of the *purMCD* mutation was carried out in both strains by introduction of the *Francisella*-*Escherichia coli* shuttle vector pFNLTP6 [17] harboring the wild-type *purMCDN* operon. Introduction of this plasmid completely restored growth of both LVS Δ*purMCD* and Schu S4 Δ*purMCD* mutants on Chamberlain's defined medium, confirming that the auxotrophic phenotype was due to loss of *purMCD* (data not shown).

### Growth of Schu S4 Δ*purMCD* is attenuated in human macrophages and lung epithelial cells

**Figure 1:**
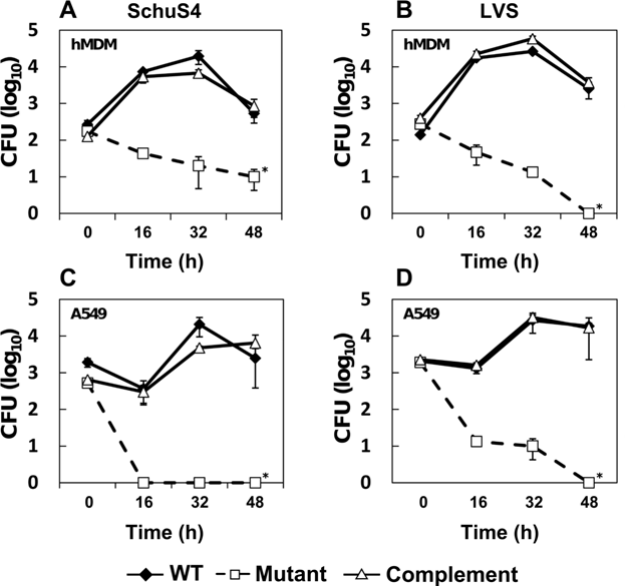
Growth of *F. tularensis* derivatives in mammalian cells. Intracellular growth of *F. tularensis* Schu S4 and LVS derivatives in human monocyte-derived macrophages (A and B), and A549 lung epithelial cells (C and D). Cells were infected with wild type *F. tularensis* LVS or Schu S4, LVS or Schu S4 Δ*purMCD*, or the LVS or Schu S4 genetically complemented Δ*purMCD*, and intracellular growth was monitored by lysing cells at the indicated time points and determining CFU by standard plate count. Infections were conducted at an MOI of 1, and gentamicin (5 µg/ml) was added to culture medium after infection to kill extracellular organisms. Standard errors of organ burden from three individual mice are shown. Asterisks indicate significance between wild-type or complemented Δ*purMCD* mutants and Δ*purMCD* burdens (*P*<0.05; two-way ANOVA).

Macrophages are a primary target for *F. tularensis* during mammalian infection and are generally thought to contain limiting concentrations of purines [18], [19]. Previous characterization of the LVS *purMCD*::Km^r^ mutant indicated that this strain was able to infect murine macrophages and escape the host cell phagosome prior to lysosomal fusion, but was unable to persist within the host cell cytosol and was cleared shortly thereafter [16]. Schu S4 Δ*purMCD* demonstrated a similar attenuation in both murine peritoneal-derived primary macrophages and the macrophage-like cell line J774A.1 (data not shown). To determine whether the unmarked Schu S4 Δ*purMCD* mutant was also attenuated for growth in human cells, human peripheral blood monocyte-derived macrophages (hMDM) were infected with this derivative at a multiplicity of infection (MOI) of 1, and bacterial burden determined every 16 hours for two days. Schu S4 Δ*purMCD* was significantly attenuated (*P*<0.01) in hMDMs relative to the wild-type parent over the time course examined (Fig. 1A). Complementation with wild-type *purMCDN in trans* restored this growth defect. In addition to macrophages, *F. tularensis* has also been shown to associate with human alveolar type II lung epithelial cells [20]. Therefore, growth of mutant *F. tularensis* derivatives was also assessed in the A549 cell line. Growth of Schu S4 Δ*purMCD* was significantly attenuated relative to the wild-type and complemented strains in these cells (*P*<0.05) (Fig. 1C). Similar results were also observed in unmarked Δ*purMCD* mutant and complemented derivatives of LVS that were generated in parallel (Fig. 1B and D). Thus, the inability of type A or type B *F. tularensis* to synthesize purines *de novo* results in a severe growth defect in primary human macrophages and cultured human lung epithelial cells.

### Schu S4 Δ*purMCD* is attenuated for virulence in mice

Previous studies of the LVS Δ*purMCD*::Km^r^ mutant indicated that this strain was highly attenuated for virulence in mice when delivered via the i.p. infection route [16]. To determine whether the unmarked Δ*purMCD* mutants were attenuated *in vivo* when delivered via more relevant routes of infection, groups (n = 5) of six-to-eight week-old BALB/c mice were infected intranasally (i.n.) or intradermally (i.d.) with 10-fold serial dilutions (ranging from 10 CFUs to 10^6^ CFUs) of wild-type, Δ*purMCD*, or genetically complemented Schu S4 or LVS mutant derivatives. Even at the lowest dose tested, all mice succumbed to infection with either wild-type Schu S4 or the complemented Schu S4 Δ*purMCD* mutant with mean times-to-death (MTD) of between 6–12 days. Thus, the observed LD_50_ for these strains is <10 CFUs (Table 1). The higher MTD observed in the complemented Schu S4 Δ*purMCD* mutant compared to wild-type Schu S4 is most likely a consequence of plasmid instability or loss due to the inability to maintain selection *in vivo*. In contrast, all animals infected either i.n. or i.d. with Schu S4 Δ*purMCD* displayed no overt disease symptoms, and all animals survived infection for the 21-day duration of the experiment (Table 1). Therefore, the LD_50_ of the Schu S4 Δ*purMCD* mutant is >10^6^ CFUs. A similar pattern of results was also observed in mice infected via either infection route with the analogous LVS Δ*purMCD* mutant and complemented derivatives (data not shown). Thus, deletion of *purMCD* in type A *F. tularensis* or in LVS results in severe virulence attenuation in the intradermal and intranasal murine infection models.

**Table 1:** Virulence of Schu S4 derivatives in BALB/c mice.

Strain	Route	Survival^a^	LD_50_ a ^,^ b	MTD (days)^a^
Schu S4	i.n.	0/5	<10	6.6
	i.d.	0/5	<10	7.6
Schu S4 Δ*purMCD*	i.n.	5/5	>10^6^	>21
	i.d.	5/5	>10^6^	>21
Schu S4 Δ*purMCD*∶pTZ752	i.n.	0/5	<10	6.8
	i.d.	0/5	<10	11.6

a Survival, LD_50_, and mean time-to-death (MTD) were determined 21 days after infection for the lowest Schu S4 and Schu S4 Δ*purMCD*∶pTZ752 doses (10 CFU) given, and the highest Schu S4 Δ*purMCD* dose (10^6^ CFU).

b LD_50_ values were calculated as described by Reed and Muench.

### Schu S4 Δ*purMCD* is cleared in mice shortly after infection

**Figure 2:**
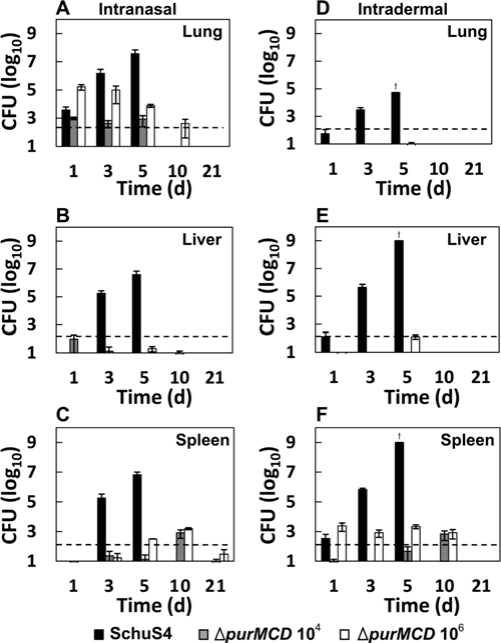
Growth characteristics of Schu S4 derivatives in BALB/c mice. Groups of mice were infected i.n. (A, B, and C) or i.d. (D, E, and F) with wild-type Schu S4 (50 CFU), or one of two doses of Schu S4 Δ*purMCD* (10^4^ or 10^6^ CFU). At specific times after infection, subsets of mice were sacrificed and the numbers of bacteria present in the lungs (A and D), livers (B and E), or spleens (C and F) were determined by plating organ homogenates on MH agar containing ampicillin (50 µg/ml). Values represent the mean log CFU per ml from groups of three animals each. The horizontal dashed line represents the confidence level of detection for the assay. Standard errors of organ burdens for three individual mice are shown. Crosses indicate time points where n = 1; n = 3 for all other time points.

To determine whether Schu S4 Δ*purMCD* persisted in target organs after infection, groups (n = 3) of age-matched mice were infected i.n. or i.d. with either 50 CFUs of wild-type Schu S4, or 10^4^ or 10^6^ CFUs of Schu S4 Δ*purMCD* and bacterial burdens monitored in the lung, spleen, and liver over a 21 day period. Mice inoculated with wild-type Schu S4 showed a rapid increase in CFUs in target organs regardless of the route of infection, and all animals died by 6 days post-infection (Fig. 2). In contrast, Schu S4 Δ*purMCD* failed to replicate to high numbers in target organs, and was eventually cleared. However, mutant organisms were detectable for at least ten days post-infection (Fig. 2). Burdens were highest in the lung following i.n. infection (Fig. 2A), with low levels of organisms detected in the liver and spleen at the various time points examined (Fig. 2B and C). Following i.d. infection, Schu S4 Δ*purMCD* was found predominantly in the spleen (Fig. 2F), but failed to disseminate to or survive in the lung and liver even in animals receiving the highest dose (Fig. 2D and E). Thus, Schu S4 Δ*purMCD* persists in host tissues for up to 10 days following infection, and its pattern and level of persistence is dependent upon the route of infection and the relative dose delivered.

### Vaccination with Schu S4 Δ*purMCD* increases the survival probability in mice challenged intranasally with wild-type Schu S4

**Figure 3:**
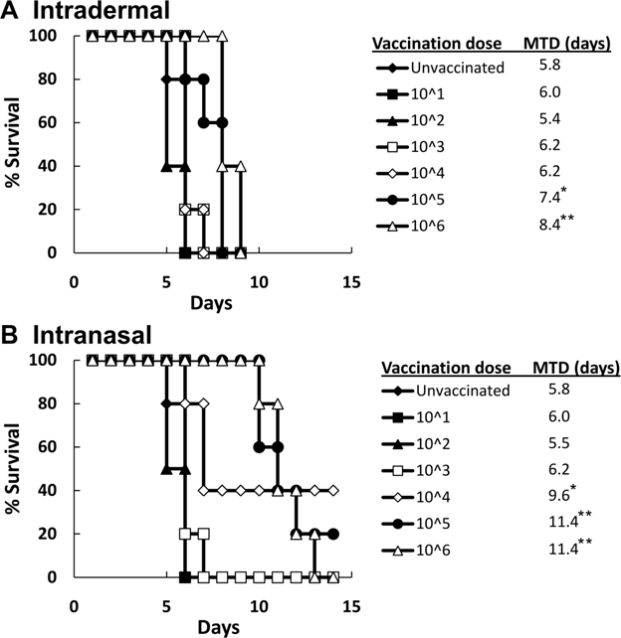
Survival of mice vaccinated with Schu S4 Δ*purMCD* and challenged i.n. with 500 CFUs of wild-type Schu S4. Mice (n = 5) were vaccinated i.d. (A) or i.n. (B) with 10-fold serial dilutions of Schu S4 Δ*purMCD* ranging from 10 to 10^6^ CFUs. 21 days post-vaccination, mice were challenged i.n. with 500 CFUs of wild-type Schu S4. The survival of mice was then monitored for 14 days. Vaccination dose is listed in CFUs, and mean time-to-death was calculated for each dose. Asterisks indicate vaccinating doses at which MTD was significant compared to unvaccinated mice (*P<0.05, **P<0.001 by log-rank analysis).

To determine whether vaccination with Schu S4 Δ*purMCD* provided protective immunity against subsequent i.n. challenge with wild-type type A *F. tularensis*, groups of age-matched mice (n = 5) were left unvaccinated or were vaccinated i.d. or i.n. with increasing doses of Schu S4 Δ*purMCD*. Mice were then challenged 21 days later i.n. with a moderate dose (500 CFUs) of Schu S4. All naïve mice infected i.d. or i.n. with wild-type Schu S4 succumbed to infection and exhibited a MTD of 5.8 days (Fig. 3A and B). Both i.d. (Fig. 3A) and i.n. (Fig. 3B) vaccination with Schu S4 Δ*purMCD* increased the overall survival of infected mice over the naïve controls based on a trend test for survival probabilities (*P*<0.0001) [21]. I.d. vaccination of mice with increasing doses of Schu S4 Δ*purMCD* enhanced the MTD from 5.8 days to 8.4 days, while the MTD in mice vaccinated i.n. with escalating doses of the mutant increased from 5.8 days to 11.4 days. Log-rank analysis confirmed that both routes of vaccination significantly increased the MTD over the naïve controls. Although all mice vaccinated i.d. with Schu S4 Δ*purMCD* eventually succumbed to infection following challenge with Schu S4 (Fig. 3A), several mice vaccinated i.n. with higher doses of Schu S4 Δ*purMCD* survived this challenge (Fig. 3B). Taken together, these results demonstrate that vaccination with Schu S4 Δ*purMCD* enhances protection against respiratory challenge with type A *F. tularensis*, with the greatest protection seen following vaccination by the intranasal route.

### Intranasal vaccination with Schu S4 Δ*purMCD* protects mice at levels that are comparable to that seen with LVS or a LVS Δ*purMCD* mutant

To date, mice vaccinated with attenuated type A *F. tularensis* mutants have failed to confer protective immunity that is better than that seen with LVS [12], [13]. To gauge the extent of protection conferred by Schu S4 Δ*purMCD*, i.n. vaccination-challenge studies were conducted in parallel with wild-type LVS and an LVS Δ*purMCD* mutant. Groups (n = 3) of age-matched mice were vaccinated i.n. with 10^2^ CFUs of wild-type LVS, 10^6^ CFUs of LVS Δ*purMCD*, or 10^4^ CFUs of Schu S4 Δ*purMCD*, or were inoculated with PBS (sham control). A vaccination dose of 10^4^ CFUs of Schu S4 Δ*purMCD* was chosen based on the results presented in Figure 3. Animals were then challenged 42 days post-vaccination with moderate (1–1.5×10^2^ CFUs) or high (2–3×10^3^ CFUs) doses of virulent type A (Schu S4) or type B (NR-648) *F. tularensis* via either the i.n. or i.d. route. In addition, half of the vaccinated animals were boosted with the respective strain 21 days post-vaccination to assess the affect of multiple immunizations on protective immunity. Boosting doses included 2×10^3^ CFUs of LVS, 1×10^6^ CFUs of Schu S4 Δ*purMCD*, or 1×10^6^ CFUs of LVS Δ*purMCD*. No CFUs were detected in the lung, spleen, or liver of any of the vaccinated mice immediately prior to challenge (data not shown). Naïve mice succumbed to infection with Schu S4 or NR-648 within 5–7 days regardless of the challenge route or dose (Table 2, rows 25–28). In contrast, mice vaccinated with a single dose of the Schu S4 Δ*purMCD* mutant were well protected against both moderate and high dose i.d. challenge with Schu S4 or NR-648 (Table 2, rows 17–20). However, protection from a single dose Schu S4 Δ*purMCD* vaccination was not notably better than in naïve animals when challenge strains were delivered via the i.n. route (Table 2, rows 17–20). Administration of a boost 21 days post-vaccination increased the protective efficacy of Schu S4 Δ*purMCD*, but these animals still remained susceptible to high dose i.n. challenge with Schu S4 (Table 2, rows 21–24). Mice vaccinated with a single dose of LVS were well protected against i.d. infection with both moderate and high doses of Schu S4 and NR-648 (Table 2, rows 1–4), and against moderate doses of Schu S4 when administered via the i.n. route (Table 2, rows 1–4). Inclusion of a booster LVS immunization provided only a minimal increase in protection against high dose Schu S4 or NR-648 i.n. challenge (Table 2, rows 5–8). Finally, mice vaccinated with a single dose of LVS Δ*purMCD* were as susceptible to i.d. or i.n. Schu S4 and NR-648 challenge as unvaccinated controls (Table 2, rows 9–12). However, protection was increased to levels comparable to that seen with Schu S4 Δ*purMCD* and wild-type LVS following administration of a booster immunization (Table 2, rows 13–16). For all strains tested, statistical analysis of data revealed a clear trend of increasing survival probability upon vaccination and vaccination followed by boosting (*P*<0.0001). In addition, administration of a boosting dose at 21 days post-vaccination collectively enhanced survival probability (*P*<0.0001). Though able to offer significant protection in most scenarios, none of the vaccine strains tested offered substantial protection against high i.n. doses of Schu S4. Thus, after boosting, all strains tested exhibited similar vaccine efficacy when comparing vaccinated to naïve mice.

**Table 2:** Vaccination and Challenge results.

Row	Vacc.	+/− Boost	Challenge	Intradermal		Intranasal	
				Survival	MTD (days)^a^	Survival	MTD (days)^a^
1	LVS (200 CFU)	−	Schu S4 (100 CFU)	3/3	>21.0	3/3	>21.0
2		−	Schu S4 (2000 CFU)	3/3	>21.0	0/3	7.0
3		−	NR-648 (150 CFU)	3/3	>21.0	1/3	15.0
4		−	NR-648 (3000 CFU)	3/3	>21.0	1/3	15.0
5		+	Schu S4 (100 CFU)	3/3	>21.0	3/3	>21.0
6		+	Schu S4 (2000 CFU)	3/3	>21.0	1/3	11.0
7		+	NR-648 (150 CFU)	3/3	>21.0	3/3	>21.0
8		+	NR-648 (3000 CFU)	3/3	>21.0	1/3	16.0
9	LVS Δ*purMCD* (10^6^ CFU)	−	Schu S4 (100 CFU)	0/3	8.3	0/3	6.0
10		−	Schu S4 (2000 CFU)	0/3	7.3	0/3	5.0
11		−	NR-648 (150 CFU)	0/3	8.7	0/3	7.7
12		−	NR-648 (3000 CFU)	1/3	11.7	0/3	7.0
13		+	Schu S4 (100 CFU)	3/3	>21.0	3/3	>21.0
14		+	Schu S4 (2000 CFU)	3/3	>21.0	1/3	16.7
15		+	NR-648 (150 CFU)	3/3	>21.0	3/3	>21.0
16		+	NR-648 (3000 CFU)	3/3	>21.0	2/3	19.0
17	Schu S4 Δ*purMCD* (10^4^ CFU)	−	Schu S4 (100 CFU)	2/3	16.3	1/7	10.7
18		−	Schu S4 (2000 CFU)	3/3	>21.0	0/3	6.3
19		−	NR-648 (150 CFU)	2/3	16.3	0/3	9.0
20		−	NR-648 (3000 CFU)	3/3	>21.0	2/3	16.3
21		+	Schu S4 (100 CFU)	3/3	>21.0	5/7	18.0
22		+	Schu S4 (2000 CFU)	3/3	>21.0	0/3	6.7
23		+	NR-648 (150 CFU)	3/3	>21.0	3/3	>21.0
24		+	NR-648 (3000 CFU)	2/3	16.7	2/3	18.0
25	None		Schu S4 (100 CFU)	0/3	6.7	0/3	5.0
26			Schu S4 (2000 CFU)	0/3	5.7	0/3	5.0
27			NR-648 (150 CFU)	0/3	6.7	0/3	7.0
28			NR-648 (3000 CFU)	0/3	6.3	0/3	6.3

a MTD, mean time-to-death was calculated for a 21-day period.

### I.n. vaccination with Schu S4 Δ*purMCD* fails to limit growth of wild-type Schu S4 in the lungs of infected mice

**Figure 4:**
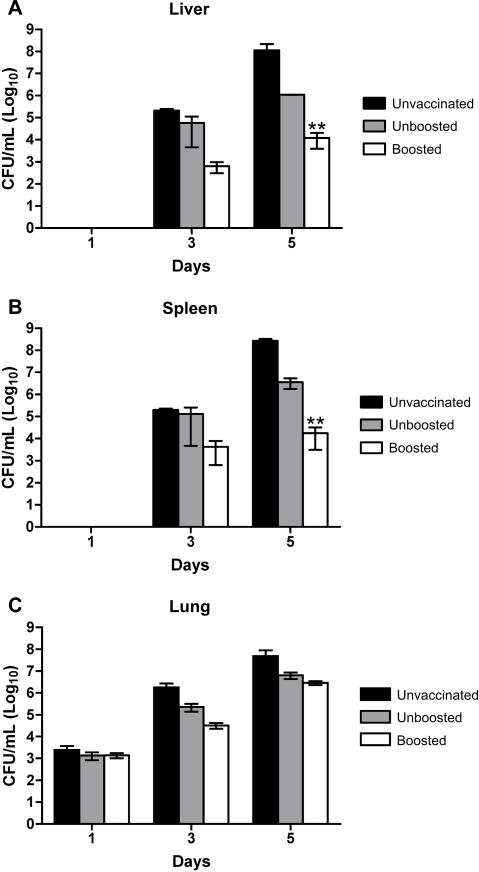
Bacterial burden in mice vaccinated i.n. with Schu S4 Δ*purMCD* and challenged with wild-type Schu S4. Mice were unvaccinated, vaccinated (10^4^ CFUs), or vaccinated (10^4^ CFUs) and boosted (10^6^ CFUs) with Schu S4 Δ*purMCD*. 42 days post-vaccination, mice were challenged with 100 CFUs of wild-type Schu S4. All vaccinations and challenges were administered by the i.n. route. At one, three, and five days post-challenge, liver, spleen, and lungs from infected animals were aseptically removed and homogenized, and a standard plate count performed on MH containing Amp. The mean and standard errors of CFUs from 3 animals per time point is shown. Asterisks indicate a significantly reduced bacterial burden compared to unvaccinated animals (*P*<0.001 by two-way ANOVA).

To determine whether i.n. vaccination with Schu S4 Δ*purMCD* limited growth of wild-type Schu S4 in the lung, bacterial burdens were determined in the lung, spleen, and liver of mice (n = 3) at one, three, and five days following i.n. challenge with 100 CFUs of Schu S4. Mice vaccinated and boosted with Schu S4 Δ*purMCD* contained significantly reduced bacterial burdens in their liver (*P*<0.01) and spleen (*P*<0.01) by 5 days post-challenge (Fig. 4A and B). In contrast, no significant reduction in bacterial burden was observed in the lungs of Schu S4 Δ*purMCD*-vaccinated and boosted mice at this time over the unvaccinated controls (Fig. 4C). Mice receiving a single vaccination with Schu S4 Δ*purMCD* did not exhibit a significant reduction in bacterial burden in the lung, spleen, or liver following Schu S4 challenge. These results indicate that mice vaccinated i.n. with Schu S4 Δ*purMCD* are less able to limit Schu S4 proliferation in the lungs compared with the liver and spleen following i.n. challenge.

### Intranasal vaccination with Schu S4 Δ*purMCD* induces regions of acute inflammation in lung tissue

**Figure 5:**
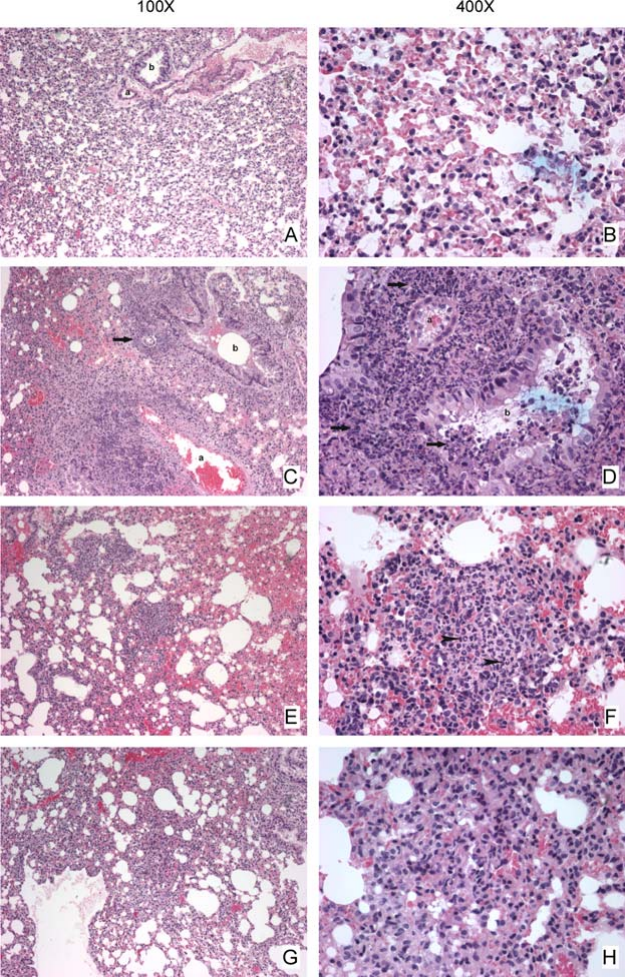
Histopathology of lungs from mice infected with Schu S4 derivatives. Mice were infected i.n. with 1× PBS (A, B), 100 CFUs of wild-type Schu S4 (C, D), or 10^6^ CFUs of Schu S4 Δ*purMCD* (E, F). Infections were allowed to proceed for 5 days before animals were euthanized and lungs removed for histology. A subset of animals was also vaccinated i.n. with 10^4^ CFUs of Schu S4 Δ*purMCD* and boosted i.n. 21 days later with 10^6^ CFUs Schu S4 Δ*purMCD*. Animals were then administered 1× PBS 42 days post-vaccination (21 days post-boost) and euthanized 4 days later for histological analysis of lung tissues (G, H). Images were captured at 100× (A, C, E, G) or 400× (B, D, F, H) magnification. Representative arteries (a) and bronchioles (b) are labeled. Examples of neutrophils are indicated by black arrowheads and necrosis by black arrows.

The ability to limit growth of the challenge strain in the spleen and liver but not in the lungs following i.n. vaccination with Schu S4Δ*purMCD* suggested that the lung may be compromised in its ability to control infection following administration of Schu S4Δ*purMCD*. To assess the type of damage inflicted on the lung after i.n. vaccination with Schu S4 Δ*purMCD*, lung tissues from individual representative uninfected mice or mice infected i.n. with wild-type Schu S4 or the Schu S4 Δ*purMCD* mutant were examined 5 days post-infection. A representative image of the lung from an uninfected animal is shown for comparison (Fig. 5A and B). Lungs from mice infected i.n. with WT Schu S4 show pronounced areas of hemorrhage in the airspaces at this timepoint (Fig. 5C and D). In addition, there are large foci of necrosis associated with bronchioles and arteries, with pockets of necrotic cellular debris within bronchiolar airspaces involving approximately 35% of the lung tissue examined (Fig. 5C and D). Although lungs from mice infected i.n. with a single dose of 10^6^ CFUs of Schu S4 Δ*purMCD* (Fig. 5E and F) do not show the same extensive regions of necrosis as seen in Schu S4-infected animals, multiple small foci of acute inflammation can be observed along with areas of neutrophil influx within the lung parenchyma affecting less than 20% of the total lung tissue. Though the acute foci of inflammation are no longer evident following vaccination (10^4^ CFUs) and boosting (10^6^ CFUs) with Schu S4 Δ*purMCD* in the lungs of the three representative mice examined, there are areas of consolidation involving between 20–30% of the lung tissue with hemmorhage and rare foci of mixed cellular infiltrates suggestive of granuloma formation (Fig. 5G and H). This indicates potential chronic disease pathology resulting from vaccination alone. Thus, i.n. vaccination with Schu S4 Δ*purMCD* induces foci of damage in the lungs of mice which may be detrimental to production of proper lung immunity in response to virulent SchuS4 challenge.

## Discussion

*F. tularensis* is a highly infectious bacterium, with as few as ten inhaled organisms able to cause severe disease in humans [2], [4]. Its extreme infectivity and capacity to cause debilitating and possibly fatal illness has warranted its classification by the United States Centers for Disease Control and Prevention as one of six Category A Select Agents most likely to be used as a biological weapon. In light of recent bioterrorism concerns, the lack of a licensed vaccine has prompted a renewed interest in generating a vaccine able to protect against infection with highly virulent *F. tularensis* biotypes.

In this study, the utility of an unmarked Schu S4 deletion mutant as a live vaccine candidate was assessed. The *purMCD* locus encodes three enzymes (phosphoribosylaminoimidazol synthetase, SAICAR synthetase, and phosphoribosylamine-glycine ligase) required for the *de novo* synthesis of purine nucleotides. Deletion of these genes abrogates the ability of *F. tularensis* Schu S4 to replicate in human macrophages and epithelial cells *in vitro*. Though unable to persist in host cells *in vitro*, *purMCD* mutants of *F. tularensis* remain able to escape from the host cell phagosome prior to fusion with the lysosome, where they persist in the host cytosol before being cleared [16]. The ability to escape from the phagosome may be an important consideration in developing a live attenuated vaccine for *Francisella*. Mutants unable to escape from the phagosome, such as those defective for IglC production, are attenuated for virulence, but fail to confer protective immunity against virulent *F. tularensis* challenge [12]. While the exact reasons for this are unclear, escape of *Francisella* into the cytosol is important for activation of host innate immune recognition systems, particularly the inflammasome, which is known to be crucial to host defenses against intracellular pathogens [22]. Phagosomal escape has also been shown to be important for processing and release of IL1-β, a cytokine that plays a key role in initiating and maintaining the pro-inflammatory response necessary for *Francisella* immunity [23]. In addition, phagosome escape and bacterial persistence in the cytosol is likely to be important for MHC Class I presentation of important antigens [24]. The inability of the Schu S4 Δ*purMCD* to replicate *in vitro* also correlates to a significant reduction in virulence *in vivo*. The LD_50_ values for these strains are ≥10^6^-fold higher than that seen with the wild-type or complemented mutant derivatives which have an observed LD_50_<10 CFUs.

A growing body of evidence indicates that the ideal live attenuated *Francisella* vaccine should be amenable to delivery by the respiratory route to promote optimal protective immunity against virulent type A *F. tularensis* delivered aerogenically [10], [11], [25]. While i.d. vaccination with high doses of Schu S4 Δ*purMCD* enhances the MTD of animals challenged i.n. with type A *F. tularensis*, all i.d. vaccinated animals eventually succumb to infection following challenge. This is likely a result of the limited dissemination of the Schu S4 Δ*purMCD* mutant to the lung following vaccination via this route. I.n. vaccination of mice with Schu S4 Δ*purMCD* is capable of providing protection against subsequent i.d. challenge with virulent type A and type B *F. tularensis*; however, protection against these strains remains limited when the challenge strains are delivered by the i.n. route. The extent of protection observed is similar to that seen following vaccination with LVS or LVS Δ*purMCD*. Though able to partially limit the dissemination and/or survival of wild-type Schu S4 in the liver and spleen, i.n. vaccination with Schu S4 Δ*purMCD* fails to appreciably limit growth of the challenge strain within the lungs. One possible explanation for this is that administration of high doses of Schu S4 Δ*purMCD* i.n. induces tissue damage in the lungs, thus limiting the ability to subsequently clear large doses of the challenge organism. Although Schu S4 Δ*purMCD* is deleted for components of the purine biosynthetic operon and is unable to proliferate within the lungs, this strain is expected to otherwise express all of the virulence determinants necessary for successful infection. In addition, respiratory infection with virulent type A strains is known to result in a robust pro-inflammatory response within the lungs of mice [26]. Histological examination of lungs from mice vaccinated with Schu S4 Δ*purMCD* show multiple foci/pockets of acute inflammation within the lung parenchyma and associated airways, along with very small foci of necrosis. Thus, inflammation resulting from challenge with wild-type Schu S4, in addition to any loss of tissue integrity following vaccination with Schu S4 Δ*purMCD*, may limit the ability of these animals to control infection in the lung. Regardless, studies are currently underway to investigate alterations in lung architecture and the immune response elicited following vaccination with Schu S4 Δ*purMCD*, LVS Δ*purMCD*, or wild-type LVS.

It has been widely speculated that the limitations of LVS as a live vaccine strain may be countered by construction of an attenuated type A derivative that carries a single or multiple defined non-reverting mutations. In theory, this would account for any antigenic differences between the two subspecies, and/or differences in the cell types encountered during infection. Comparison of the protective efficacy provided by the strains utilized in this study indicates that Schu S4 Δ*purMCD* offers no better protection than LVS as a live vaccine. This suggests that the inability of LVS to confer protection is not likely due to antigenic variation between the subspecies. In addition, vaccinating and boosting with LVS Δ*purMCD* conferred protection at levels that were comparable to that seen with wild-type LVS, suggesting that deletion of the *purMCD* locus does not adversely affect expression of important antigenic determinants. Given these observations, and the retained ability of the Schu S4 Δ*purMCD* mutant to induce tissue damage in the lungs following i.n. vaccination even in the absence of bacterial proliferation, we speculate that introduction of the Δ*purMCD* allele into an otherwise wild-type strain from the less pathogenic type B biovar may result in an attenuated derivative that could provide the necessary immunogenicity without the associated reactogenicity. Recent advances in the area of *F. tularensis* genetics should allow for mutants to be readily made in this genetic background. Regardless, further work is still needed to characterize the protective response(s) elicited by vaccination with attenuated mutants of type A or type B *F. tularensis*, and define the antigenic determinants responsible for stimulating this response.

## Materials and Methods

### Bacterial strains and culture conditions

*F. tularensis* LVS was a gift from Karen Elkins. *F. tularensis* NR-648 is a virulent type B strain and was a kind gift from Rick Lyons. *F. tularensis* Schu S4 was obtained from BEI Resources. All work with NR-648 or Schu S4 and its derivatives were conducted under BSL3 or ABSL3 containment, and were approved by the Institutional Biosafety Committees or Institutional Animal Use and Care Committees of the Medical College of Wisconsin (MCW) or The Ohio State University (OSU). MCW and OSU are registered with the Centers for Disease Control and Prevention to work with virulent strains of *F. tularensis*. *F. tularensis* strains were grown aerobically at 37°C in modified Mueller-Hinton (MH) broth or agar (Difco) supplemented with 1% proteose peptone, 2.5% fetal bovine serum (Invitrogen), 0.25% ferric pyrophosphate, 1% dextrose, and 2% IsoVitalex. When required, kanamycin (10 µg/ml), ampicillin (50 µg/ml), or sucrose (10%) was added to media. Bacterial inocula for animal infection studies was generated by streaking *F. tularensis* strains onto MH plates, resuspending bacteria in 1× PBS, and diluting to the appropriate concentration. All cloning was performed in *Escherichia coli* DH5α. *E. coli* was grown at 37°C in Luria-Bertani (LB) broth or agar. LB was supplemented with 50 µg/ml kanamycin when required.

### Allelic replacement and complementation

Unmarked deletion mutations were generated in *F. tularensis* LVS and Schu S4 using a strategy similar to that described previously [16]. Briefly, 1.0-kb regions flanking *purMCD* were amplified from *F. tularensis* LVS and directionally cloned into suicide plasmid pTZ699 [16]. A *groE-aph* kanamycin resistance cassette was then cloned into the unique *Sma*I site present in the backbone of pTZ699 to generate pTZ866. This plasmid was then electroporated into wild-type *F. tularensis* LVS or Schu S4 and transformants selected on MH containing kanamcin. Integration of the plasmid into the homologous region on the *F. tularensis* chromosome was confirmed by PCR. Merodiploids were then spread or struck on MH medium containing 10% sucrose to enrich for derivatives that had undergone a second recombination event and resolved the plasmid containing the kanamycin resistance determinant. Resulting clones were patched onto Chamberlain's defined medium (CDM) and MH containing kanamycin to confirm auxotrophy and loss of antibiotic resistance, respectively. Mutants were confirmed by PCR and by Southern blot analysis. Complementation of the Δ*purMCD* allele in both LVS and Schu S4 was accomplished by introduction of pTZ752 as described previously [16].

### Isolation and infection of macrophages and lung epithelial cells

Human peripheral blood monocyte-derived macrophages (hMDMs) were isolated from buffy coats obtained from the BloodCenter of Southeastern Wisconsin. Buffy coats were layered onto Histopaque (Sigma) gradients to enrich for lymphocytes and monocytes. Resulting pellets were washed twice with ice cold PBS containing 0.05% BSA, suspended in RPMI 1640 (Gibco), and monocytes further enriched by layering onto Percoll (GE Healthcare) gradients. Resulting cells were suspended in RPMI 1640 complete medium containing 2 mM L-glutamine and 20% human serum (Gemini Bioproducts), transferred into Teflon-coated tissue culture cups (Savillex), and incubated for 5 days to allow for differentiation of monocytes into macrophages. Macrophages were seeded into 12-well tissue culture plates at a density of 1×10^5^ cells per well and allowed to adhere overnight. Non-adherent cells were removed by washing with RPMI 1640 prior to infection with *F. tularensis*. Mouse peritoneal macrophages were obtained by injecting mice intraperitoneally with 2 ml of 2% thioglycolate (Sigma) and collecting peritoneal cells by lavage with sterile phosphate-buffered saline after 3 days. Peritoneal macrophages were maintained in Dulbecco's modified Eagle's medium (DMEM) supplemented with 10% fetal bovine serum and 2 mM L-glutamine. J774A.1 macrophages were maintained as previously described [16]. A549 human lung epithelial cells (ATCC CCL-185) were cultured in RPMI 1640 containing 5% fetal bovine serum (FBS; HyClone) and 2 mM L-glutamine. 12-well tissue culture plates were seeded at a density of 1×10^5^ for *F. tularensis* infection. All cells were maintained at 37°C in humidified air containing 5% CO_2_.

For infection of macrophages and A549 cells, *F. tularensis* strains were grown to mid-exponential phase, diluted to the appropriate concentration in pre-warmed RPMI or DMEM, respectively, and added to cells at an MOI of 1 bacterium per cell. Cells were then incubated for 2 h to allow bacterial internalization, washed with PBS, and suspended in fresh medium containing 5 µg/ml gentamicin for killing of extracellular bacteria. At specific times following infection, infected cells were washed with PBS, lysed with sterile water or 0.5% saponin (macrophages and A549 cells, respectively), and the number of CFU determined by plating serial 10-fold dilutions on MH agar medium.

### Mouse infection experiments

6- to 8-week-old female Balb/c mice (Harlan Sprague) were used in all infection studies. Mice were anesthetized prior to infection by intraperitoneal injection of 250 mg/Kg Avertin (Sigma), resulting in approximately 30 min of surgical anesthesia. For intradermal infections, mice were infected at the base of the tail with a 100 µl inoculum. Alternatively, anesthetized mice were infected intranasally with a 25 µl inoculum directly into the nares. The actual number of bacteria delivered for each infection was quantified by plating the initial inoculum on MH agar. For analysis of bacterial burden in infected mice, mice were euthanized at specific times after infection, and the lung, liver and spleen removed aseptically. Infected tissues were homogenized and diluted in PBS, and total CFU determined by plating on MH agar medium containing Amp. LD_50_ experiments were conducted essentially as described [16]. For vaccination-challenge studies, groups of mice were inoculated i.n. with 1× PBS, or vaccinated with defined doses of wild-type LVS, the Schu S4 Δ*purMCD* mutant, or the LVS Δ*purMCD* mutant. Some animals in each group received a booster immunization of the same strain given 21 days post-vaccination. 42 days after the initial vaccination, mice were challenged i.n. with moderate or high doses of wild-type Schu S4 or NR-648. All infections were allowed to proceed to the designated times or until animals become visibly moribund. Mean time-to-death was calculated by dividing the sum of the survival times of all mice by the total number of mice examined, with surviving animals given a time of 21 days, when the experiment was terminated.

### Histopathology

Histology was conducted on lungs from representative mice infected with various *F. tularensis* derivatives. At various times following vaccination and/or infection, lungs were removed aseptically and fixed in 10% zinc formalin for a minimum of 24 hours. Lung tissues were then processed and embedded in paraffin, and five-micrometer sections mounted on slides. Between 4 and 6 levels were obtained for each tissue block. Slides were stained with hematoxylin/eosin, and examined by an anatomic pathologist (N.H.S) using a Nikon E400 upright microscope. Between 10–25 10× fields were examined per tissue level. Images were captured using a SPOT camera and SPOT software version 3.5.4 (Diagnostic Instruments, Inc., USA).

### Statistical Analysis

A two-way ANOVA analysis was used to determine significance in survival between *F. tularensis* derivatives in hMDM and A549 epithelial cells (Fig. 1). A test for trend of survival probabilities [21] was applied to determine significance in survival probabilities following i.d. or i.n. vaccination with Schu S4 Δ*purMCD* and challenge with wild-type Schu S4 (Fig. 3). Before application of this test, log-rank or Wilcoxon weights were used to specify weight functions. Log-rank analysis was applied to MTD values to confirm significance relative to that seen in unvaccinated control animals. A Cochran-Armitage trend analyses [27] was used to confirm that a significant trend in survival probabilities existed among vaccinated and unboosted, and vaccinated and boosted groups relative to naïve mice in vaccination/challenge studies (Table 2). A proportional hazards model (Cox model) was used to model survival times, where vaccinating strains were compared for boost effect and vaccine vs. control effect [21]. Finally, for analysis of bacterial burdens in mice following Schu S4 challenge in unvaccinated, vaccinated, and vaccinated and boosted mice (Fig. 4), a log transformation with base ten was applied to the data before analysis by two-way ANOVA. All values were considered significant at *P*<0.05, and highly significant at *P*<0.001. Methodological limitations included small sample sizes limiting the power of the tests, and reliability of the effect estimation (hazard ratio+regression coefficients).
